# Forced Fox-P3 expression can improve the safety and antigen-specific function of engineered regulatory T cells

**DOI:** 10.1016/j.jaut.2022.102888

**Published:** 2022-10

**Authors:** Jenny McGovern, Angelika Holler, Sharyn Thomas, Hans J. Stauss

**Affiliations:** a Institute of Immunity and Transplantation, Division of Infection and Immunity, University College London, Royal Free Hospital, London, UK; b Quell Therapeutics, 84 Wood Lane, London, UK

## Abstract

Regulatory T cells (Treg) are potent inhibitors of autoreactive T cells. The intracellular transcription factor FoxP3 controls the expression levels of a diverse set of genes and plays a critical role in programming functional Tregs. Although, antigen-specific Tregs are more potent than polyclonal Tregs in treating ongoing autoimmunity, phenotype plasticity associated with loss of FoxP3 expression in Tregs can lead to the conversion into antigen-specific effector T cells which might exacerbate autoimmune pathology. In this study, we designed a retroviral vector driving the expression of FoxP3 and a human HLA-DR-restricted TCR from the same promoter. Transduction of purified human Tregs revealed that all TCR-positive cells had elevated levels of FoxP3 expression, increased CD25 and CTLA4 expression and potent suppressive function. Elevated FoxP3 expression did not impair the *in vitro* expansion of engineered Tregs. Adoptive transfer into HLA-DR transgenic mice revealed that FoxP3+TCR engineered Tregs showed long-term persistence with stable FoxP3 and TCR expression. In contrast, adoptive transfer of Tregs engineered with TCR only resulted in the accumulation of TCR-positive, FoxP3-negative T cells which displayed antigen-specific effector function when stimulated with the TCR-recognised peptides. Our data indicate that forced expression of FoxP3 can prevent accumulation of antigen-specific effector T cells without impairing the engraftment and persistence of engineered Tregs.

## Highlights

• Tregs require Fox-P3
• Forced Fox-P3 locks Treg phenotype.
• Forced Fox-P3 prevents Teff conversion.

## 1 Introduction

Regulatory T cells (Treg) are potent suppressors of the immune response and are required for the maintenance of tolerance to self-tissues in humans and in mice [[1], [2], [3]]. Transfer of high doses of activated and expanded polyclonal Tregs has shown potential therapeutic efficacy in various preclinical murine models of autoimmunity and transplantation [[4], [5], [6], [7], [8], [9], [10], [11], [12], [13], [14]], which has facilitated ongoing and completed clinical trials of adoptive Treg therapy in patients [15]. Promising clinical results with polyclonal Tregs were obtained in patients after HLA-mismatched hematopoietic stem cell transplantation [16,17]. In this setting, the frequency of Tregs recognising allogeneic HLA molecules is expected to be as high as 1–10%, which may explain the observed therapeutic efficacy. In contrast, the frequency of Tregs recognising specific antigens involved in autoimmunity is expected to be more than 1000-fold lower, which reduces their therapeutic activity substantially. Several studies in preclinical disease models have indicated an improved therapeutic efficacy of antigen-specific Tregs compared with polyclonal Tregs [[18], [19], [20], [21]]. However, the production of antigen-specific Tregs by *in vitro* stimulation of polyclonal T cells with antigen is challenging and prone to failure. This limitation can be overcome by genetic engineering platforms that readily and reliably generate high frequency of antigen-specific T cells. We and others have shown that it is possible to re-direct the specificity of Treg through genetic engineering to express novel TCRs or chimeric antigen receptors (CAR), and the engineered Tregs efficiently suppressed pathologic immune damage *in vivo* [22,23]. Therefore, the engineering of antigen-specific Tregs offers an opportunity to direct the suppressive activity to antigens present in tissues affected by autoimmunity and limit systemic immune suppression in healthy tissues.

FoxP3 is the master transcription factor of Tregs that is essential for their development and function [[24], [25], [26]]. It acts to stabilise Treg phenotype through repression of non-Treg genes including IL2 and IFNγ and promotion of Treg-associated genes such as CD25 and CTLA4. However, under conditions of repeated antigen stimulation, lymphopenia or low concentration of IL-2, loss of FoxP3 expression can result in Tregs losing suppressive activity and instead produce effector cytokines that may exacerbate disease [27]. Conversely, high expression of endogenous FoxP3 in human Treg is associated with strong suppressive function *in vivo* and *in vitro* [28], although it is not clear whether gene transfer to further elevate FoxP3 would be beneficial or detrimental for Treg function.

In this study we explored whether elevated expression of FoxP3 under the control of a constitutively active promoter resistant to physiological gene regulation was detrimental to the Treg function *in vitro*. Following adoptive transfer into HLA transgenic mice, we assessed whether engineered FoxP3 expression prevented the accumulation of FoxP3-negative effector T cells *in vivo*.

## 2 Materials and methods

### 2.1 Collection of PBMC

50 ml of blood was collected from healthy donors under the research ethics approved project: Control 08/H0720/46. PBMC were isolated using a density centrifugation protocol.

### 2.2 Vectors

**Fig. 1:**
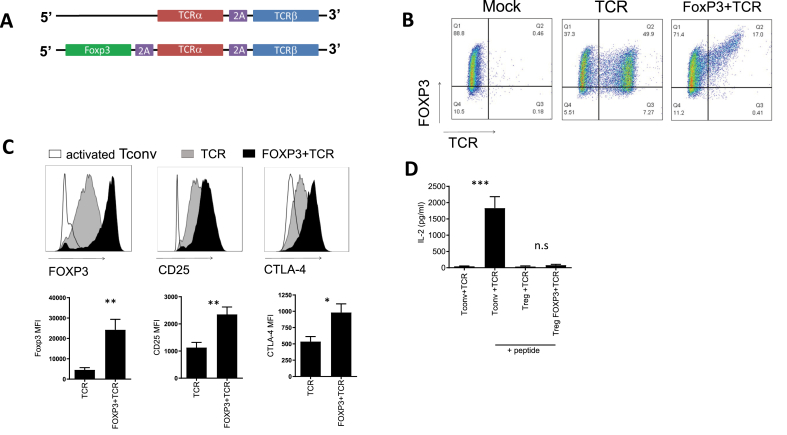
**Tregs transduced with FoxP3** + **TCR vectors express elevated levels of FoxP3, CTLA-4 and CD25. (A)** Schematic diagram of pMP71 retroviral vector encoding MS-TCR alpha and beta chains (TCR) and Foxp3 plus MS-TCR alpha and beta chains (TCR + FOXP3). (**B)** CD4^+^CD25^+^CD127^low^ Treg were isolated by FACS sorting. Treg were transduced with the vectors described in (A) or cultured with virus-free supernatant (mock). Representative flow cytometric analysis was performed at day 7 to assess level of transduction through expression of murine TCR constant regions and Foxp3. Representative of 10 independent experiments. (**C)** Representative histograms showing FoxP3, CD25 and CTLA-4 in Tregs transduced with FoxP3+TCR or TCR or control Tregs. Bar graphs summary of MFI from 13 independent experiments. Error bars show standard error of the mean. (**D)** ELISA data showing IL-2 production from transduced conventional CD4 T cells (Tconv) and transduced Treg cultured for 3 days in the presence or absence of peptide (Tconv n = 13, Treg n = 4). Error bars show standard error of the mean. *p=>0.05, **p=>0.01 determined by unpaired *t*-test.

A myelin basic protein (MBP)-specific TCR with murine constant regions was cloned into a pMP71 retroviral backbone with or without a wildtype human or mouse FOXP3 sequence (see Fig. 1A). The TCR recognises MBP (LSRFSWGAEGQRPGFGYGG) presented by HLA-DRB1*0401 and is cross reactive for mouse and human MBP.

### 2.3 Human T cell isolation and expansion protocol

CD4^+^ T cells were isolated using a positive selection kit (Miltenyi). Cells were subsequently stained with flow cytometry antibodies CD4 V500 (BD), CD25 APC Cy7 (BD) and CD127 FITC (BD) before FACS sorting using the BD ARIA. CD4^+^CD25hiCD127low Treg and CD4^+^CD25^−^CD127+ conventional T cells (Tconv) were collected. Purity of CD4^+^CD25^+^CD127lowFoxp3+ cells was routinely >70%. **Tregs:** activated 1:1 with human anti-CD3/28 Dynabeads (Gibco) in Texmacs media (Miltenyi) with 100Units/mL penicillin (Gibco); 100 μg/mL streptomycin (Gibco) without IL-2 for two days. On day two cells were supplemented with fresh media and1000u/ml IL-2 (Roche). IL-2 and fresh media were added every two days for 10 days. If indicated, at day 10 Treg were restimulated 1:1 with anti-CD3/28 dynabeads and cultured as described above until day 20. **Tconv**: were activated for 48 h by culturing 1:1 with anti-CD3 and anti-CD28 Dynabeads (Gibco) and IL-2 (Roche) at 300u/ml. Media exchange was performed on alternate days and cells were used for experiments 7 days post-transduction.

### 2.4 Flow cytometry staining

0.5x10 [6] cells were washed in PBS and stained with fixable viability dye (ThermoFisher) resuspended in PBS for 15 min. Cells were washed with FACs buffer (1% FBS in PBS) and stained with appropriate amount of fluorescent conjugated antibody diluted in FACS buffer (determined by prior titration). Intracellular staining was carried out using the Foxp3/Transcription Factor Staining Buffer Set (eBioscience).

### 2.5 Production of retroviral vectors (human and murine)

The retroviral packaging lines Phoenix-Ampho and Phoenix-Eco cells were seeded in 100 mm tissue culture-treated petri-dishes for 24 h. On day 1 cells were transfected using Fugene transfection reagent (Promega). On day 3 supernatant containing retrovirus was removed from the Phoenix cells and centrifuged at 500xG for 5 min to remove any cellular debris before use.

### 2.6 Human cell transduction

On day 2 post-activation Treg and Tconv were transduced with retrovirus on Retronectin-coated plates (Takahara-bio – Otsu, Japan). Plates were centrifuged at 800×*g* for 2 h in 32 °C. Transduction efficiency was determined by staining for murine TCRβ constant region (BD).

### 2.7 Antigen specific activation of T cells

CD80^+^ and CD86+HLA-DRB1*0401 CHO JM1 cells were mixed together and cultured with saturating amounts of peptide (10 μM/ml) for 2 h at 37 °C before being irradiated at 120Gy. Transduced T cells were plated in a 96-well u bottom plate 1:1 with CHO cells and incubated at 37°, 5% CO2 for 18–20 h. Supernatants were collected for ELISA analysis. All peptides were synthesised and supplied by ProImmune, Oxford, UK.

### 2.8 Suppression assay

Antigen-presenting CHO cells (see above) were loaded with MBP peptide and irradiated at 120Gy. The transduction efficacy of TCR-transduced Tconv and TCR-transduced Tregs was determined by flow cytometry to allow percentage of transduced cells to be normalised in the assay. Cells were plated at a starting ratio of 10 transduced Tconv: 10 transduced Treg:1 CHO cell (1:1), with decreasing numbers of transduced Treg in additional wells as indicated. The cultures were incubated at 37°, 5% CO2 for 4 days and supernatants were collected for ELISA analysis. Cell pellets were analysed by flow cytometry for CFSE dilution. Percentage suppression was analysed using the following formula: % suppression = (CFSE dilution of Tconv alone - CFSE dilution of Tconv + Tregs)/CFSE dilution of Tconv alone*100.

### 2.9 IL-2 and IFNγ ELISA

IL-2 and IFNγ level in supernatant was determined using OptEIA ELISA kits (BD – 555,148 IL-2 and 555,138 IFNγ) following the provided protocols. Samples were routinely diluted 1:5 in assay diluent and run in duplicate.

### 2.10 Retroviral transduction of murine regulatory T cells

On day 0 splenocytes and lymph nodes were resected from donor mice. Treg were isolated using the CD4^+^CD25^+^ Treg isolation kit (Miltenyi) following the manufacturers instructions and activated for 48 h by culturing 1:2 with murine anti-CD3 and anti-CD28 Dynabeads (Gibco) and 500u/ml IL-2 (Roche) in RPMI-1640 (Gibco) supplemented with 10% heat inactivated foetal bovine serum (Biowest); 100Units/mL penicillin; 100μg/mLstreptomycin; 2 mM l-glutamine (Gibco); 100 μM 2-mercaptoethanol (Gibco). On day 2 Tregs were transduced with retrovirus and placed into culture in a final concentration of 500u/ml IL-2. Cells were incubated for 24 h at 37°, 5% CO_2_ before supplementing with fresh media and 500u/ml IL-2. After an additional 24 h cells were prepared for injection. Transduction efficiency was determined by staining for human TCRβ 2.1 (Invitrogen).

### 2.11 In vivo engraftment

C57BL/6 HLA-DR4 transgenic mice (Taconic) were conditioned by irradiation with 4Gy. 4 h later mice were injected intravenously with congenically marked (CD45.1 or Thy1.1) autologous Tregs transduced with either TCR or TCR + FOXP3 1:1 (based on FACS staining for transduction efficiency). After 7 weeks mice were sacrificed and lymphoid organs harvested to examine engraftment. Tissues were mechanically digested and treated with ACK lysis buffer as required. Tissue samples were pooled and a CD4^+^ T cell isolation kit (Miltenyi) was used to enrich CD4^+^ T cells. The entire CD4^+^ fraction was stained for human TCR, FOXP3 and Treg markers.

### 2.12 Antigen specific activation of splenocytes

CD80^+^CD86 + DR4+ CHO cells were loaded with peptide and irradiated at 120gy. Enriched CD4^+^ T cells from *in vivo* engraftment experiments were plated in a 96-well u-bottom plate 1:1 with CHO cells in the presence of 2 μg/ml of Brefeldin A (Sigma). Cells were incubated at 37°, 5% CO2 for 18–20 h and for flow cytometry analysis with *anti*-IL2 and *anti*-IFNγ.

### 2.13 Data analysis

Flow Cytometry Analysis – FlowJo (Flowjo,LLC). Statistical Analysis – Graphpad Prism v.5 (Graphpad, Software).

## 3 Results

### 3.1 Transduction of treg with TCR and exogenous FoxP3 reinforces a treg phenotype

We designed a construct that encoded for a TCR recognising human myelin basic protein (MBP) peptide_111-129_ (LSRFSWGAEGQRPGFGYGG) presented by HLA-DRB1*0401. We also designed a second construct with an additional FoxP3 gene preceding the MBP-specific TCR, referred to as ‘FoxP3+TCR’ (Fig. 1A). The human TCR sequence was modified to contain murine constant α and β domains which improves expression in human T cells and facilitates analysis of TCR expression using antibodies specific for the murine constant β domain [29]. FACS sorted human CD4^+^CD25^+^CD127^low^ Tregs were transduced and expression of the introduced TCR and FoxP3 was used to determine transduction efficiency on day 7 (Fig. 1B). Higher percentage of transduced cells was seen with TCR alone compared to FoxP3+TCR, which is probably due to the larger insert in the retroviral vector reducing viral titre. However, Treg transduced with FoxP3+TCR had consistently higher levels of FoxP3 compared to Treg transduced with TCR alone (Fig. 1B and C). Moreover, Treg transduced with FoxP3+TCR had significantly increased expression of CD25 and CTLA-4 as determined by mean fluorescence intensity (Fig. 1C). Treg transduced with FoxP3+TCR or TCR were stimulated with MBP peptide pulsed antigen presenting cells to demonstrate lack of IL-2 production by engineered Tregs compared with the robust IL2 production by conventional CD4 T cells engineered with the same TCR (Fig. 1D).

### 3.2 FoxP3 transduced treg expand *in vitro* and retain phenotype

**Fig. 2:**
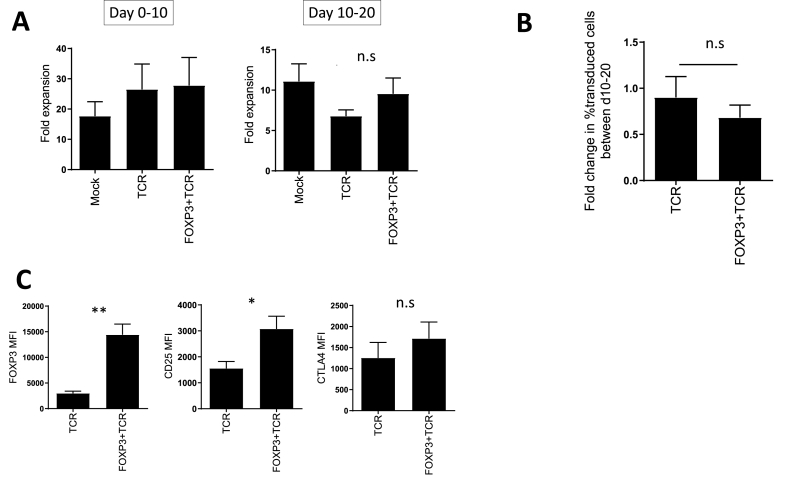
**Transduced Treg expand in vitro and retain phenotype.** Treg were activated with anti-CD3 and anti-CD28 beads with IL-2 for 10 days, beads were removed and replaced with fresh beads and IL-2 for a further 10 days. (**A)** Cells were counted at d0, d10 and d20. Left panel shows relative expansion of Treg between d0 and d10 (n = 13). Right panel shows relative expansion between d10 and d20 (n = 5). Error bars show standard error of the mean. Statistical significance determined by one-way anova. (**B)** The percentage of transduced Tregs was determined at d10 and d20. The graph shows the change in the percentage of transduced Tregs (n = 5). Error bars show standard error of the mean. Statistical significance determined by unpaired *t*-test **C.** Graphs show summary of MFI of FoxP3, CD25 and CTLA-4 of transduced Tregs at day 20 (n = 5). Error bars show standard error of the mean. *p=>0.05, **p=>0.01 determined by unpaired *t*-test.

Next, we assessed whether elevated expression of FoxP3 impaired the expansion potential of gene engineered Tregs *in vitro*. Following stimulation with CD3/CD28 beads in the presence of IL-2, the numbers of Treg were determined after 10 days, and after an additional stimulation cycle at day 20. We saw no difference in the expansion of Treg after 10 and 20 days comparing non-transduced cells (mock) with those transduced with TCR or FoxP3+TCR (Fig. 2A). Following expansion between day 10 and 20, the proportion of transduced Tregs remained similar in TCR and FoxP3+TCR engineered cells, indicating that the exogenous FoxP3 did not impair *in vitro* expansion of Tregs (Fig. 2B). Analysis of the levels of FoxP3, CD25 and CTLA-4 at d20 after *in vitro* expansion revealed that expression of these Treg markers remained elevated in cells transduced with FoxP3+TCR compared to TCR alone (Fig. 2C).

### 3.3 Treg transduced with FoxP3+TCR are more suppressive than treg transduced with TCR alone

**Fig. 3:**
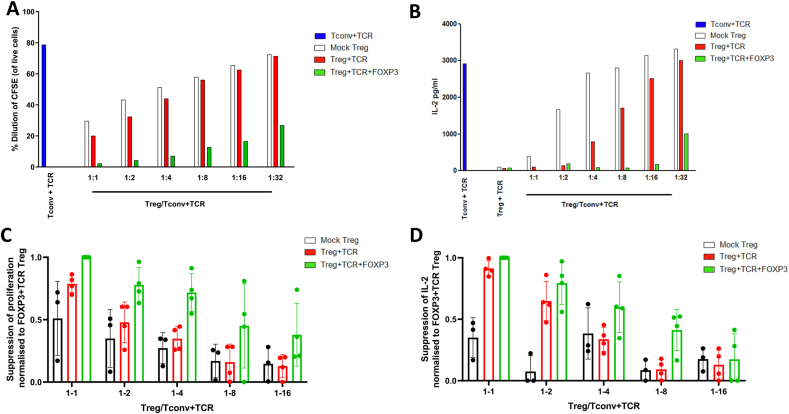
**TCR-transduced Treg suppress T cell responses in an antigen-specific manner**. TCR transduced conventional CD4 T cells (T-conv, blue bars) were stained with CFSE and cultured with or without peptide-pulsed irradiated APC at a ratio of 1 Tconv:0.01 APC for 4 days with Treg added at the indicated ratios (**A**) Dilution of CFSE-stained Tconv in the absence or presence of mock Treg (white bars), TCR-transduced Treg (red bars) or FoxP3+TCR transduced Treg (green bars) was determined by flow cytometry (**B**) Supernatant from cells cultured in (A) were collected and assayed for IL-2 by ELISA. (**C, D**) Summary data showing suppression of CFSE-dilution (C) or IL-2 production (D) of Tconv + TCR by mock Treg (black n = 3) or TCR-transduced Treg (red n = 4) or FoxP3+TCR transduced Treg (green n = 4). Shown is the relative suppression compared to the maximal suppression seen at the 1:1 ratio of FoxP3+TCR Treg to Tconv, which was set at 1.

We tested whether elevated FoxP3, CD25 and CTLA-4 expression in Treg transduced with FoxP3+TCR was associated with improved suppressive function. Conventional CD4^+^ T cells were transduced with the MBP-specific TCR and stimulated with MBP peptide-pulsed APCs in the presence of reducing numbers of Treg transduced with FoxP3+TCR, TCR only or mock transduced controls. We found that TCR-transduced Treg were better suppressors of proliferation (Fig. 3A) and IL-2 production (Fig. 3B) than mock-transduced Tregs. However, the most effective suppression of proliferation and IL-2 production was achieved with Treg transduced with FoxP3+TCR. Superior suppression of proliferation (Fig. 3C) and IL-2 production (Fig. 3D) by FoxP3+TCR transduced cells was consistently seen in 4 independent experiments using Tregs from 4 different donors.

### 3.4 Elevated FoxP3 expression prevents treg conversion into effector T cells *in vivo*

**Fig. 4:**
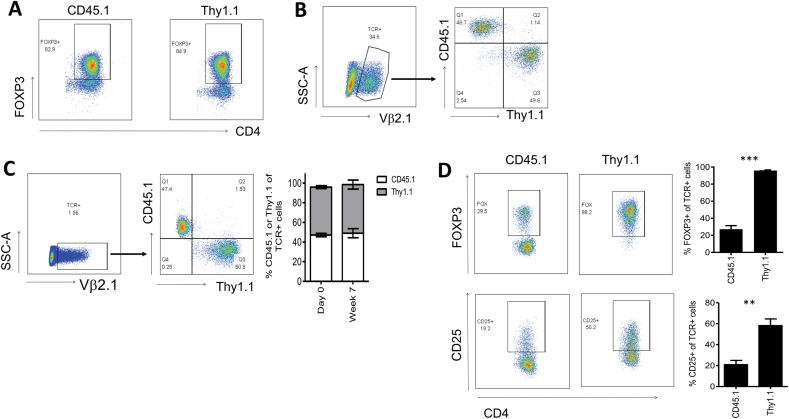
**Treg transduced with FoxP3** + **TCR are protected from conversion into FoxP3-negative T cells following engraftment in vivo**. Treg were purified from the spleen and lymph nodes of naïve CD45.1 and Thy1.1 mice using CD4 and CD25 as markers. CD45.1+ cells were transduced with TCR only and Thy1.1 cells were transduced with FoxP3+TCR before injection at a 1:1 ratio into HLA-DRB1*0401/Thy1.2/CD45.2+ recipient mice conditioned with 4Gy irradiation. (**A**) FoxP3 purity of cells isolated from CD45.1 Thy1.1 mice. (**B**) Analysis of the CD45.1/Thy1.1 cell mixture injected into HLA-DRB1*0401/Thy1.2/CD45.2+ recipients. FACS plots show that transduced human Vb2^+^ cells contained similar numbers of CD45.1 (TCR only transduced) and Thy1.1 (FoxP3+TCR transduced) cells. (**C**) After 7 weeks mice were euthanized and transduced CD4 T cells were identified by staining with anti-human Vb2 antibodies (left panel). The Vb2^+^ cell population contained similar numbers of CD45.1 (TCR only transduced) and Thy1.1 (FoxP3+TCR transduced) cells (right panel). The bar graph shows the mean percentage TCR-expressing CD45.1 (white bars) and Thy1.1 (grey bars) at day 0 and week 7 (n = 5). Error bars show standard error of the mean. (**D**) FACS plots show FoxP3 and CD25 expression profiles of TCR-expressing cells in the gated CD45.1 population (left panel) and the gated Thy1.1 population (right panel). The bar graph shows mean percentage FoxP3 or CD25 in TCR-expressing CD45.1 or Thy1.1 cells in 5 different mice. Error bars show standard error of the mean. **P=<0.001, ***P=<0.0001 determined by unpaired T test.

In order to test whether elevated levels of exogenous FoxP3 affected *in vivo* persistence and phenotype stability of engineered Tregs, we purified CD4^+^CD25^+^ murine Treg from congenic CD45.1+ or Thy1.1+ C57Bl/6 donors and transduced them with the human HLA-DRB1*0401-restricted TCR only, or with FoxP3+TCR. Fig. 4A shows similar purity of the Treg populations isolated from Thy1.1 and CD45.1 donor mice. Following retroviral gene transfer, the transduced Tregs were identified by staining with antibodies against human Vβ2, the variable region used by the HLA-DRB1*0401-restricted TCR (Fig. 4B). CD45.1+ Treg transduced with the TCR only, and Thy1.1 Treg transduced with FoxP3+TCR were mixed at a 1:1 ratio (Fig. 4B) and then injected into HLA-DRB1*0401 transgenic C57Bl/6 mice that were conditioned with 4 Gy irradiation. After 7 weeks we identified the transferred cells in the spleen by staining with anti-human Vβ2 antibodies (Fig. 4C) and found that in all mice analysed the ratio of CD45.1 and Thy1.1 cells remained very close to the 1:1 ratio seen prior to the adoptive transfer (Fig. 4C). However, while more than 98% of the Thy1.1 Tregs engineered with FoxP3+TCR retained high levels of FoxP3, more than 70% of the CD45.1 Tregs engineered with TCR only had lost FoxP3 expression (Fig. 4D).

**Fig. 5:**
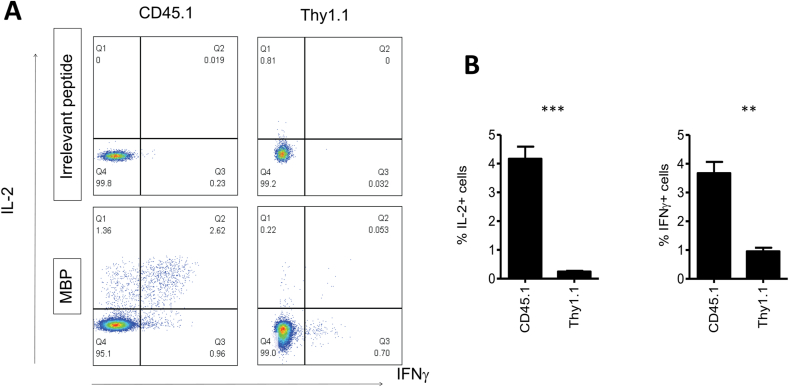
**FoxP3 expression prevents conversion into effector T cells.** Seven weeks after *in vivo* transfer, the CD4^+^ splenocytes characterised in Fig. 4 were cultured with HLA-DRB1*0401-positive cells pulsed with human MBP peptide or control peptide for 4 h in Brefeldin A before staining with CD45.1, Thy1.1, IL-2 and IFNγ. FACS plots show IL-2 and IFNγ staining of gated CD45.1 cells or Thy1.1 cells in response to irrelevant peptide or MBP peptide. The bar graph shows mean percentage of IL-2+ cells IFNγ+ cells in CD45.1+ or Thy1.1+ cells from 3 different mice. Error bars show standard error of the mean. **P=<0.001, ***P=<0.0001 determined by unpaired T test.

Finally, we determined whether the loss of FoxP3 expression in the Tregs from CD45.1 donors was associated with the gain of effector function. Hence, we stimulated total CD4^+^ splenocytes with HLA-DRB1*0401 positive antigen presenting cells pulsed with the TCR-recognised peptide of MBP. Gating on CD45.1+ cells and Thy1.1+ cells combined with intracellular cytokine staining allowed us to determine peptide-specific IL-2 and IFNγ production in Tregs transduced with TCR only and Fox-P3+TCR, respectively. Fig. 5A and B shows robust peptide-specific IL-2 and IFNγ production in gated CD45.1 cells in all mice, while the Thy1.1 cells displayed minimal cytokine production. These results indicated that exogenous FoxP3 expression substantially reduced the risk of TCR engineered Tregs to convert into antigen-specific effector T cells *in vivo*.

## 4 Discussion

In this report we demonstrate that engineered expression of FoxP3 in TCR-transduced Treg can prevent accumulation of antigen-specific effector cells without reducing Treg engraftment and long-term persistence *in vivo*. In addition, we found that engineered Treg expressing elevated levels of FoxP3 displayed enhanced suppression of T cell effector function *in vitro*.

High levels of CD25 and CTLA4 expression in murine Treg is associated with potent suppressive activity *in vivo* [30]. Similarly, high expression of FoxP3, CD25 and CLTA-4 in humans was found in a highly suppressive subset of activated Treg. Clinical studies revealed an association between the *in vivo* accumulation of FoxP3high Treg, and the induction of stable tolerogenic microenvironments [31]. However, the functional consequence of constitutive supraphysiological FoxP3 expression in Tregs was unknown. We demonstrated here that forced high expression of FoxP3 resulted in elevated CD25 and CTLA4 expression in transduced Tregs, and that the elevated FoxP3, CD25 and CTLA4 expression was associated with enhanced suppression function *in vitro*. Importantly, the supraphysiological FoxP3 expression was not associated with reduced Treg expansion *in vitro* or with impaired engraftment and persistence *in vivo*.

The potential plasticity of Treg raises a distinct safety concern for the clinical application of antigen-specific Treg therapy. The loss of FoxP3 in transferred Treg could result in the accumulation of antigen-specific effector T cells capable of aggravating autoimmunity. Here, we showed that the transfer of Tregs transduced with TCR alone, resulted in the accumulation of FoxP3-negative effector T cells that produced IL-2 and IFNγ in response to cognate peptide stimulation. In contrast, adoptive transfer of Treg transduced with FoxP3+TCR resulted in efficient long-term engraftment of cells that retained FoxP3 expression and showed negligible IL-2 and IFNγ after peptide stimulation. The data indicated that our retroviral vector driving FoxP3 and TCR expression ‘locked’ all engineered cells into functionally stable Tregs which do not produce effector cytokines. It is likely that two mechanisms are operating to ensure a ‘locked’ Treg function. Firstly, forced FoxP3 expression can counteract the downmodulation of endogenous FoxP3 which can lead to the accumulation of FoxP3-negative effector T cells. Secondly, forced FoxP3 expression may convert contaminating conventional CD4 T cells that are present in the purified Treg populations used for genetic engineering. Previous studies from our group and others have shown that forced FoxP3 expression readily converted conventional CD4 T cells into Treg-like cells that can mediate suppression *in vitro* and, following adoptive transfer, *in vivo* [23]. Taken together, the FoxP3+TCR vector platform provides a double safety mechanism for the production therapeutic Treg by converting contaminating conventional CD4 T cells into Treg-like cells, and by counteracting the consequences of endogenous FoxP3 downmodulation that can lead to the accumulation of effector CD4 T cells. Although the relative importance of each mechanism is unknown, our data show that together the two mechanisms are effective in preventing the *in vivo* accumulation of substantial populations of antigen-specific effector CD4 T cells seen when FoxP3 is absent in the gene transfer vector.

## 5 Conclusion

We propose that engineering Treg to express high levels of exogenous FoxP3 provides a double safety feature of adoptive Treg therapy, while also improving the suppressive function of the engineered cell product.

## Author statement

We confirm that the manuscript has been read and approved by all named authors and that there are no other persons who satisfied the criteria for authorship but are not listed. We further confirm that the order of authors listed in the manuscript has been approved by all of us.

## Declaration of competing interest

JM is currently and employee of Quell Therapeutics. HJS is a co-founder and adviser of Quell Therapeutics, shareholder of Kuur Therapeutics and adviser of PanCancerT.

## Data Availability

Data will be made available on request.
